# Nowhere to Invade: *Rumex crispus* and *Typha latifolia* Projected to Disappear under Future Climate Scenarios

**DOI:** 10.1371/journal.pone.0070728

**Published:** 2013-07-29

**Authors:** Zhonglin Xu, Zhaodong Feng, Jianjun Yang, Jianghua Zheng, Fang Zhang

**Affiliations:** 1 College of Resource & Environmental Science, Xinjiang University, Urumqi, China; 2 Key Laboratory of City Intellectualizing and Environment Modelling, Xinjiang University, Urumqi, China; 3 Xinjiang Institute of Ecology and Geography, Chinese Academy of Science, Urumqi, China; The Ohio State University, United States of America

## Abstract

Future climate change has been predicted to affect the potential distribution of plant species. However, only few studies have addressed how invasive species may respond to future climate change despite the known effects of plant species invasion on nutrient cycles, ecosystem functions, and agricultural yields. In this study, we predicted the potential distributions of two invasive species, *Rumex crispus* and *Typha latifolia*, under current and future (2050) climatic conditions. Future climate scenarios considered in our study include A1B, A2, A2A, B1, and B2A. We found that these two species will lose their habitat under the A1B, A2, A2A, and B1 scenarios. Their distributions will be maintained under future climatic conditions related to B2A scenarios, but the total area will be less than 10% of that under the current climatic condition. We also investigated variations of the most influential climatic variables that are likely to cause habitat loss of the two species. Our results demonstrate that rising mean annual temperature, variations of the coldest quarter, and precipitation of the coldest quarter are the main factors contributing to habitat loss of *R. crispus*. For *T. latifolia*, the main factors are rising mean annual temperature, variations in temperature of the coldest quarter, mean annual precipitation, and precipitation of the coldest quarter. These results demonstrate that the warmer and wetter climatic conditions of the coldest season (or month) will be mainly responsible for habitat loss of *R. crispus* and *T. latifolia* in the future. We also discuss uncertainties related to our study (and similar studies) and suggest that particular attention should be directed toward the manner in which invasive species cope with rapid climate changes because evolutionary change can be rapid for species that invade new areas.

## Introduction

Species invasion has contributed to the extinction of native species [1], alteration of fire regimes [2], nutrient cycling [3], functioning of ecosystems [4], economic losses [5], reduction of agricultural yield [6], spreading of diseases [7], and gene pollution [8]. Controlling the growth and spread of invasive species is expensive. In addition, adaptive responses of invasive species to global climate change may result in more complex and robust invasion mechanisms in the long run. Given such uncertainty, a detailed understanding of the effect of climate change on invasive species is very important.

Generally, an invasive species may respond to climate change in two ways. First, a species can expand its geographic distribution in several areas to find more suitable climatic conditions [9], [10]. By contrast, their geographic distribution can shrink in some areas to avoid unfavorable conditions [11]. Distributions of invasive species are limited by climatic condition at global and regional scales. As such, regardless of whether these species expand or shrink their geographic distributions, an insight into their response to climatic variables, which is at the core of the invasion process, is essential [12].

Species distribution modeling is a valuable approach for understanding the relationship between the presence of a species and climatic conditions [13]. This approach allows determination of the relationship between the presence of a target species and climatic features of the locations they inhabit. By applying the relationship to a wider geographic range, a researcher can obtain the potential geographic distribution of any given species. By applying the relationship to different climate scenarios (past or future), the projected (past or future) potential invasion area of a species can thus be modeled. More than 10 species distribution models can currently be used to predict the potential invasion areas of target species. These models can be classified into two categories based on their data requirement: (1) models that require only presence data for prediction, and (2) models that require both presence and absence data for prediction [14]. Reliable absence data for a species are generally difficult to collect. As a result, models that require only presence data are valuable. These models include BIOCLIM [15], HABITAT [16], DOMAIN [17], genetic algorithm for rule-set prediction [18], ecological niche factor analysis [19], Mahalanobis distance [20], and maximum entropy (MaxEnt; [21]). Among these models, MaxEnt is reported to outperform others and has been widely used in studies related to species invasion [11], [22], [23], [24], [25], [26].

In the present study, two plant species, namely *Rumex crispus* and *Typha latifolia*, were selected to investigate the effect of climate change on species invasion [27], [28]. These species were selected because of their wide invasion ranges and harmful effects to native species and ecosystems worldwide. We first predicted the potential distributions of *R. crispus* and *T. latifolia* under current climatic conditions. Then, the potential distributions of these two species under future climatic conditions (five scenarios) were modeled. Correlation between climate change and the invasion mechanism of these two species was studied by comparing different potential distributions under different climatic conditions. Our findings improve our understanding of the effect of climate change on species invasion of R. crispus and T. latifolia and suggest that our approach may be broadly applicable to the study of other plant species as well.

## Materials and Methods

### Species

**Figure 1:**
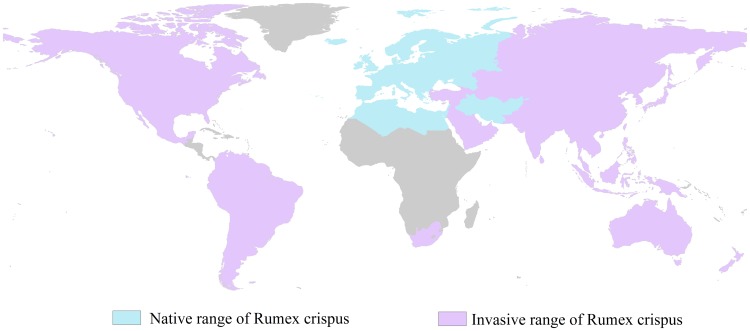
Native and invasive range of *R. crispus*.

*R. crispus,* also known as curled dock, is native to Europe, northern Africa (i.e., Algeria, Egypt, Libya, Morocco and Tunisia), and western Asia (i.e., Afghanistan, Iran, Iraq, Israel, Lebanon; [29]). Figure 1 shows the native range of *R. crispus*. This species grows in a wide variety of habitats, including disturbed soil, waste areas, roadsides, fields/meadows, shorelines, and forest edges and prefers rich, moist, and heavy soil in general. This species can be used as a wild leaf vegetable because its leaves are an excellent source of vitamin A, protein, iron, and potassium. *R. crispus* is propagated through the contamination of crop seeds and by sticking to clothing. It is classified as an “injurious weed” under the United Kingdom Weed Act of 1959 (http://www.defra.gov.uk/farm/wildlife/weeds/). As a widespread naturalized species throughout the temperate world, *R. crispus* is now present in continental Asia, Japan, North and South America, North and South Africa, Australia, and New Zealand [29],and is considered as one of the five most widely distributed plants in the world (see Figure 1 for invasive range).

**Figure 2:**
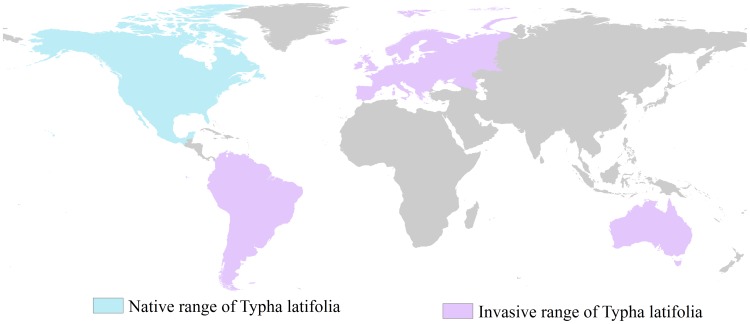
Native and invasive range of *T. latifolia*.

*T. latifolia* is a perennial herbaceous plant that belongs to the genus Typha. It is a native plant species of North America (ranging from Alaska to Guatemala, as shown in Figure 2; [30]). *T. latifolia* grows in a variety of climates, including tropical, subtropical, southern and northern temperate, humid coastal and dry continental. This species is found at elevations ranging from 0 m.s.l. to 2300 m.s.l. As an obligate wetland species, *T. latifolia* is always found in or near water. It generally grows in flooded areas where the water level does not exceed 0.8 m. Traditionally, *T.* *latifolia* has been part of several native North American cultures as a source of food and medicine. Their rhizomes are edible after cooking and removing the skin. Similarly, their young flower spikes are edible. Several cultures use the roots of *T. latifolia* as a poultice for boils, burns, or wounds. This species often plays important roles in keeping lakes healthy by filtering runoffs. *T.* *latifolia* forms dense monocultures when a wetland disturbance occurs. It can reach up to 3 m height and can grow prolifically from thick underground rhizomes, forming dense rhizome mats and litter that may reduce the chance of survival or spreading of other plants. The invasion range of *T. latifolia* is shown in Figure 2.

### Data

Presence samples and environmental data are necessary in order to estimate the potential distribution ranges of a plant species. In the present study, presence samples of *R. crispus* and *T.* *latifolia* were obtained from the biodiversity data portal Global Biodiversity Information Facility (GBIF, www.gbif.org). We collected a total of 61,772 and 35,262 presence samples of *R.* *crispus* and *T. latifolia*, respectively. Duplicate samples may have been included (because of the resolution of environmental layers) in this data set, so we used a sample selection strategy that excluded duplicated samples (only one sample at each pixel). In addition, such a huge number of presence samples may induce overfitting. Therefore, the presence samples data obtained from GBIF must be filtered. Fortunately, the model used in this study (MaxEnt, described in the following section) fulfills this requirement. Environmental data on current climatic conditions were obtained from the WorldClim Web site (http://www.worldclim.org). The Worldclim data set includes annual time series of mean monthly data for precipitation as well as minimum and maximum temperatures recorded by more than 4,000 weather stations worldwide [31]. This data set consisted of the following 19 climatic layers: mean annual temperature, mean diurnal range, isothermality, temperature seasonality, maximum temperature of the warmest month, minimum temperature of the coldest month, temperature annual range, mean temperature of the wettest quarter, mean temperature of the driest quarter, mean temperature of the warmest quarter, mean temperature of the coldest quarter, mean annual precipitation, precipitation of the wettest quarter, precipitation of the driest quarter, precipitation seasonality, precipitation of the wettest quarter, precipitation of the driest month, precipitation of the warmest quarter, and precipitation of the coldest quarter. These layers were spatially interpolated according to the constructed relationship between recorded variables and terrain features (i.e., latitude, longitude, and elevation). Compared with other climatic data sets, the WorldClim data set exhibited the following advantages: the resolution of data layers was improved, more weather station records were used for the interpolation, and improved elevation data were used. In the present study, data layers with resolution of 2.5 arc min (approximately 5 km at the equator) were adopted. In order to predict the potential distribution of target species under future climatic conditions, future climatic layers are necessary. Future climatic conditions consisting of 19 climatic layers were downloaded from the Climate Change, Agriculture, and Food Security Web site (http://www.ccafs-climate.org). Future climatic projections included the Intergovernmental Panel on Climate Change-Special Report on Emissions Scenarios (IPCC-SRES) A1B (very rapid economic growth, global population that peaks in mid-century and declines thereafter, based on a balance across all sources), A2 (a highly heterogeneous world with continuously increasing population, economic development is primarily regionally oriented), A2A (a highly heterogeneous world with high rate of population growth, regionally oriented economies), and B1 (a convergent world with the same global population, rapid change in economic structures toward a service and information economy) for 2050 predicted by the Canadian Centre for Climate Modeling and Analysis-Third-Generation Coupled Global Climate Model (CCCMA-CGCM3), and B2A (regionally oriented economies with a general evolution towards environmental protection and social equity) for 2050 predicted by CCCMA-CGCM2 [32].

### Species Potential Distribution Modeling

The potential distributions of each species were predicted using the MaxEnt model, which was developed based on the principle of maximum entropy. Under this principle, a target probability distribution can be determined by finding the probability distribution of maximum entropy (i.e., the most spread out or the one closest to a uniform distribution; [21]), which is subject to a set of constraints representing incomplete information regarding the target distribution. When this principle is applied to predict the potential species distribution at each pixel across the study area, the constraint becomes the expected value of each environmental variable which matches the empirical average [21]. The MaxEnt model is increasingly being used to model potential species distribution and has been shown to outperform other modeling approaches because of the following advantages: (1) only presence samples are required; (2) it guarantees an optimal probability distribution through an efficient deterministic algorithm; (3) it generates an output with a concise definition and is, therefore, amenable to analysis; and (4) overfitting can be effectively avoided [21], [33]. In the present study, the model was applied using the default settings [34]. The presence samples were randomly partitioned with 80% assigned to the training dataset and the remaining 20% to the testing dataset. MaxEnt uses the presence data and randomly selected points, and combines these with environmental variables to predict probability values ranging from 0 (completely not suitable) to 1 (completely suitable) for each cell. Continuous probability values (ranging from 0 to 1) can be transformed into binary (0 for predicted unsuitable; 1 for predicted suitable) values by applying a threshold. The maximum sensitivity plus specificity (MSS) approach [35] was adopted for the selection of a threshold. The MSS approach originated from the confusion matrix, which is composed of four elements denoted by *a*(true positive, recorded present and predicted present), *b*(false positive, recorded absent but predicted present), *c*(false negative, recorded present but predicted absent), and *d*(true negative, recorded absent and predicted absent). The sensitivity and specificity values were determined by calculating 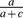 and 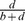, respectively. The MMS approach determined the threshold by maximizing the value of 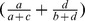 [35]. When the prediction was complete, performance of the model was evaluated using the area under the receiver operating character curve (AUC; [36]).

### Relationship between Potential Distribution of Species and Climatic Variables

As mentioned previously, investigating the response of invasive species to each climatic variable is essential to understand the effect of climate change on the invasiveness of species. The MaxEnt model can estimate the contribution of each climatic variable on the potential distribution of species. In the present study, the relationship between potential distribution of the target species and climatic variables was analyzed based on the following strategies. First, the current potential distribution areas of these two species were delineated, and the relatively important climatic variables that contributed more than 10% were determined. Second, the values of these important variables at current potential distribution areas were extracted from the current and future climatic layers to obtain two datasets, namely, VPC and VPF. Next, mean minimum, mean maximum, and global mean of each variable in VPC and VPF were calculated. Third, comparisons of the differences between each pair of variables in VPC and VPF were performed at three levels (i.e. mean minimum, mean maximum, and global mean). In this way, differences in the contribution of each variable in the same geographic region under different climatic conditions (current and future) could be detected. Given that the value of climatic variables was extracted from the same geographic regions (i.e., the current potential distribution area), presence of differences between current and future potential distributions would permit examination of the effect of variation of variables on species distribution. In this study, future climatic conditions were represented by five climate scenarios (A1B, A2, A2A, B1, and B2A). Therefore, the average value of each variable among these five scenarios was calculated to determine future climatic conditions.

## Results

We obtained probabilities of the distribution suitability of the target species using the MaxEnt model, the threshold values, which translated the probability to binary data (1 for predicted presence and 0 for predicted absence) were also determined. For *R. crispus*, the threshold value was 0.441. All pixels with values higher than 0.441 were classified as suitable for the distribution of *R. crispus*. The threshold for *T. latifolia* was 0.423. Performance of the model was evaluated by calculating the AUC value. With AUC values of 0.896 and 0.902 for *R. crispus* and *T. latifolia*, respectively, the MaxEnt model performed a reliable prediction of the potential distributions of the two species.

**Figure 3:**
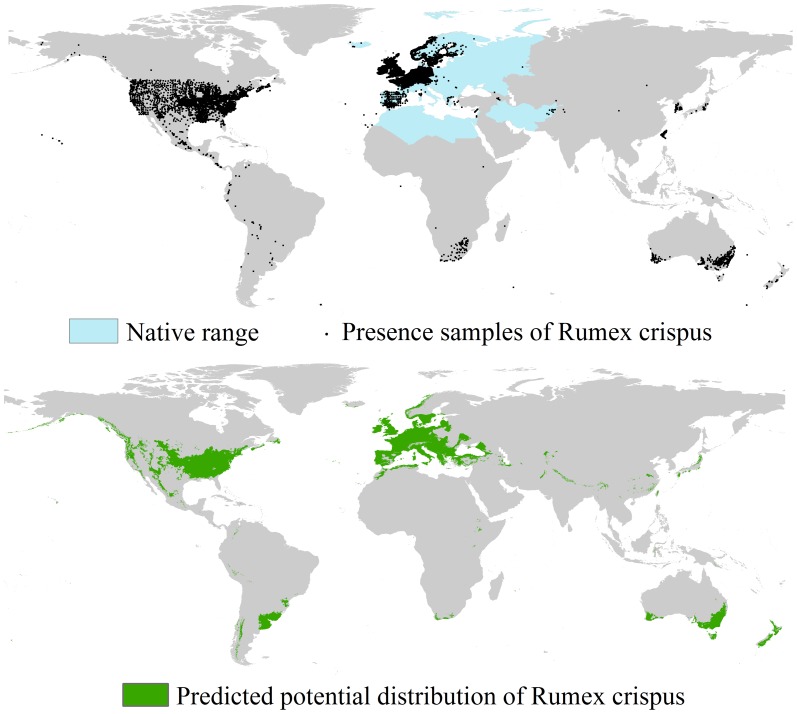
Native range, presence samples, and potential distribution of *R. crispus*. All presence samples (black dots) were used in potential distribution modeling.

As shown in the upper panel of Figure 3, the collected presence samples within the ranges indicated in blue are the native samples while those outside the blue ranges are the invasive samples. The lower panel of Figure 3 shows the potential distribution (green ranges) of *R. crispus* under current climatic conditions. The area predicted to be suitable for *R. crispus* exceeds 17 M km^2^. The potential and actual distributions of *R. crispus* are similar for North America (mainly the United States), Europe (Sweden and Norway for the northern part; Great Britain, France, Germany, and Italy for the western and central parts; and Spain and Portugal for the southern part), and Australia. The regions with different potential and actual distributions are predicted to be affected by the invasion of *R. crispus*. As shown in Figure 3, these regions include parts of Europe (Latvia, Lithuania, Belarus, Poland, Czech Republic, Slovakia, Hungary, Croatia, Serbia, Albania, Macedonia, Romania, Bulgaria, Ukraine, and Turkey), New Zealand, and parts of South America (Uruguay and Argentina). The invasion potential of *R. crispus* also exists in areas along the Himalayas, Andes, and Rocky Mountains (Figure 3).

**Figure 4:**
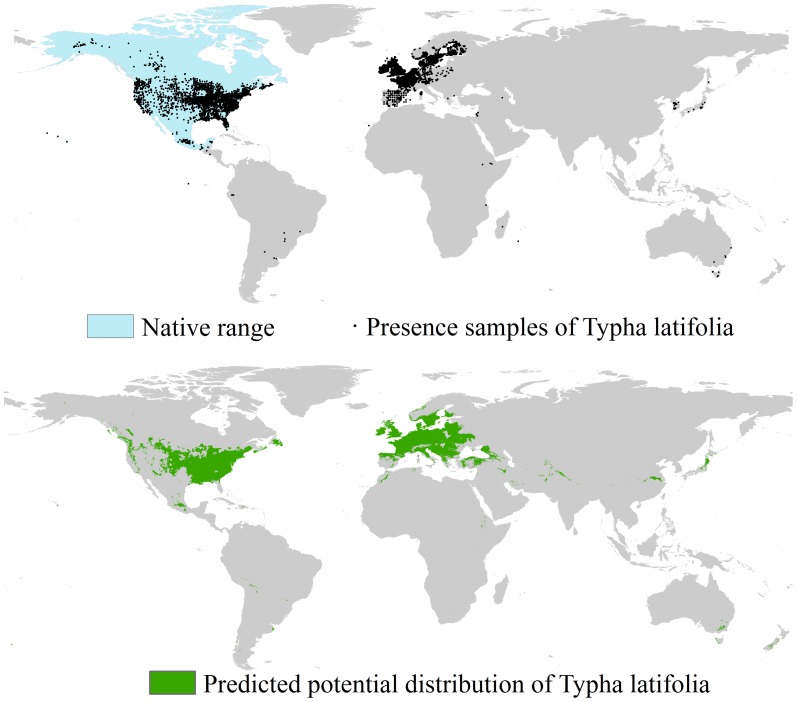
Native range, presence samples, and potential distribution of *T. latifolia*. All the presence samples (black dots) were used in potential distribution modeling.

The presence samples, native range, and potential distribution of *T. latifolia* under current climatic conditions are shown in Figure 4. As shown in the upper panel of Figure 4, the collected presence samples within the ranges indicated in blue are the native samples while those outside the blue ranges are the invasive samples. The potential distribution of the species is shown in the lower panel of Figure 4 (green ranges). The potential and actual distributions are similar in North America (Canada and the United States) and Europe (Sweden, Norway, Great Britain, France, Italy, Germany, Spain, Portugal, etc.). Risk of invasion exists in areas where the actual and potential distributions are different, such as Latvia, Lithuania, Belarus, Poland, Czech Republic, Slovakia, Hungary, Croatia, Serbia, Albania, Macedonia, Romania, Bulgaria, Ukraine, and Turkey. In addition, potential for invasion exists in several parts of China, Japan, and India (Figure 4). The area predicted to be suitable for *T. latifolia* exceeds 14 M km^2^.

**Figure 5:**
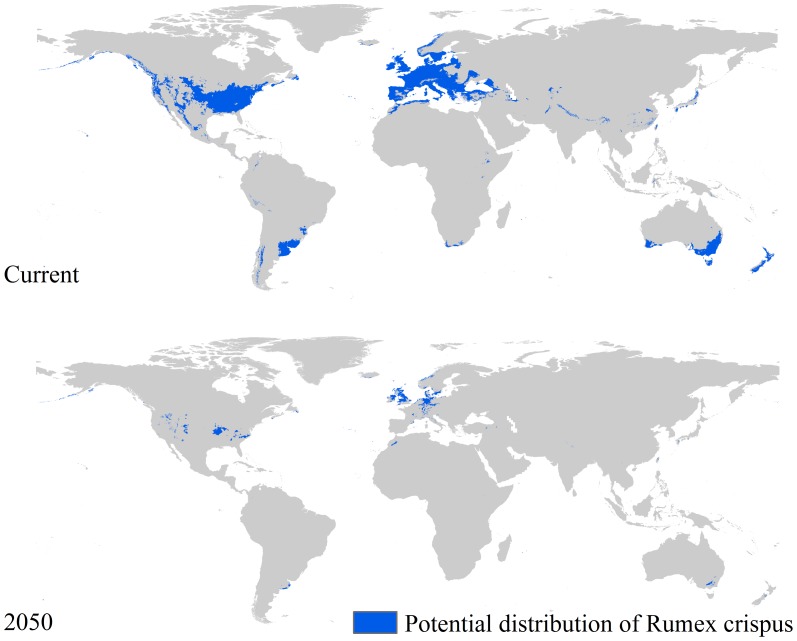
Potential distribution of *R. crispus* under climatic conditions for 2050 under the B2A scenario.

**Figure 6:**
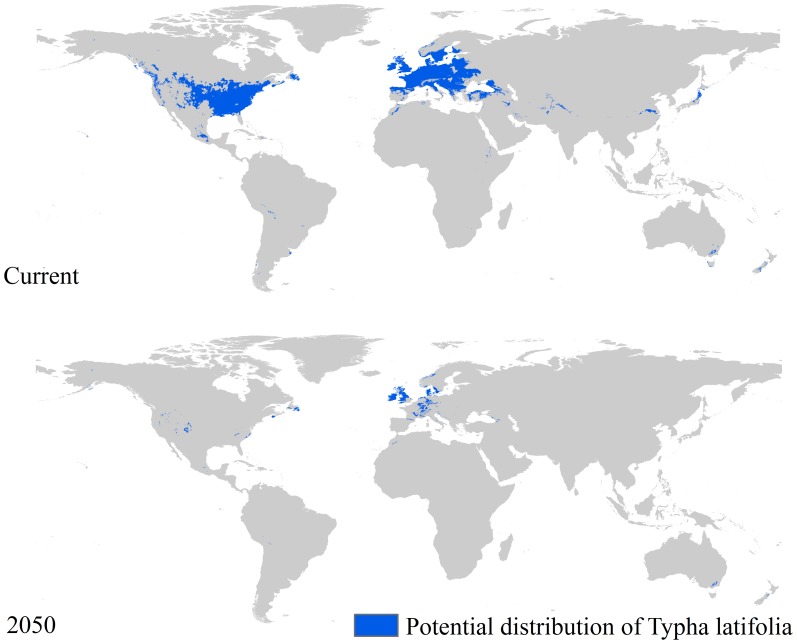
Potential distribution of *T. latifolia* under climatic conditions for 2050 under the B2A scenario.

Employing the same strategies we used to predict the potential distribution of *R. crispus* and *T. latifolia* under current climatic conditions, we predicted the potential distribution of these two species under future climatic conditions (IPCC-SRES A1B,A2, A2A, and B1 for 2050 predicted using CCCMA-CGCM3, and B2A for 2050 predicted using CCCMA-CGCM2). No pixel with a value of one was found under the A1B, A2, A2A, and B1 scenarios, this means the future climatic condition related to these four scenarios are not suitable for the distribution of these two species. Their distributions will be maintained under future climatic conditions related to B2A scenarios, but the total area will be less than 10% of that under the current climatic condition. Figure 5 and 6 clearly show the variations of such potential distributions. As shown in Figure 5, the potential distribution of *R. crispus* covers less than 160,000 km^2^ while that of *T. latifolia* covers about 130,000 km^2^.

We found that for *R. crispus*, the mean temperature of the coldest quarter, mean annual temperature, and precipitation of the coldest quarter contribute the most (more than 10%) to the potential distribution of the species (Table 1). Specifically, mean annual temperature contributed 26.3%, and mean temperature of the coldest quarter and precipitation of the coldest quarter contributed 31.7% and 16.4%, respectively, to its potential distribution. Contributions of these three variables totaled 74.4%. For *T. latifolia*, five climatic variables contributed more than 10% to the potential distribution of the species (Table 1). These variables include mean annual temperature, mean temperature of the coldest quarter, mean annual precipitation, precipitation of the driest month, and precipitation seasonality (coefficient of variation). Their contribution rates were 25.8%, 11.5%, 10.0%, 15.8%, and 13.4%, respectively, for a total contribution of 76%.

**Table 1:** Bioclimatic variables in areas predicted to be suitable for the two subject species under current climatic conditions, but unsuitable under future climatic conditions.

Most influential bioclimatic variables(% contribution)		Current mean minimum	Current global mean	Current mean maximum	Future mean minimum	Future global mean	Future mean maximum
*Rumex crispus*	MAT (°C, 26.3%)	3.31	11.64	19.83	3.05	12.20	21.69
	TCQ (°C, 31.7%)	−8.34	3.15	14.76	−9.76	5.12	15.23
	PCQ (°C, 16.7%)	34	711	927	32	709	940
*Typha latifolia*	MAT (°C, 25.8%)	0.02	9.59	18.97	3.00	12.04	21.30
	TCQ (°C, 11.5%)	−14.4	1.56	13.1	−9.81	4.96	15.73
	MAP (mm, 10.0%)	307	1457	2077	280	1580	2276
	PDM (mm, 15.8%)	4	56	97.5	4.5	66.3	107
	PS(13.4%)	8.5	68.5	97.2	8.4	74.3	102.1

Mean minimum and maximum values were an average of 5% extreme values. MAT = mean annual temperature, TCQ = mean temperature of the coldest quarter, PCQ = precipitation of the coldest quarter, MAP = mean annual precipitation, PDM = precipitation of the driest month, and PS = precipitation seasonality (coefficient of variation). MAT, TCQ, and PCQ contributed the most to the potential distribution of *R. crispus*. MAT, TCQ, MAP, PDM, and PS contributed the most to the potential distribution of *T. latifolia.*

Table 1 shows that in the current potential distribution areas of *R. crispus*, the global mean and mean maximum value of the mean annual temperature as well as the mean temperature of the coldest quarter under current climatic conditions are relatively lower than those under future climatic conditions. The mean minimum values of these two variables under current conditions are relatively higher than those under future conditions. This variation in temperature indicates that the mean value for the two variables shifts to a relatively higher value (from 11.64 to 12.20 for mean average temperature and from 3.15 to 5.12 for mean temperature of the coldest quarter) and that range becomes wider (from a range of 3.31 to 19.83 to a range of 3.05 to 21.69 for mean average temperature, and from a range of −8.34 to 14.76 to a range of −9.76 to 15.23 for the mean temperature of the coldest quarter). Table 1 also indicates that for *T. latifolia*, the mean annual temperature and the temperature of the coldest quarter under current climatic conditions are relatively lower than those under future conditions at all three levels (mean minimum, mean maximum, and global mean). This result demonstrates a warmer shift in the current potential distribution area of *T. latifolia* (the mean annual temperature shifts from a range of 0.02 to 18.97 to a range of 3.00 to 21.30, and the temperature of the coldest quarter shifts from a range of −14.4 to 13.1 to a range of −9.81 to 15.73).

The effects of precipitation-related climatic variables cannot be neglected considering their significant contribution: 16.7% contribution of the precipitation of the coldest quarter for *R. crispus*, and 10.0%, 15.8%, and 13.4% contributions of mean annual precipitation, precipitation during the driest month, and precipitation seasonality, respectively, for *T. latifolia*. For *R. crispus* in the current potential distribution areas, precipitation during the coldest quarter is slightly less at the mean minimum level under future climatic conditions compared with that under current conditions, and slightly more at the mean maximum level under future climatic conditions compared with that under current conditions. This result indicates a broader range of precipitation during the coldest quarter. For *T. latifolia*, a similar range shift to a broader range could also be detected in the mean annual precipitation (from a range of 307 to 2077 to a range of 280 to 2276). Precipitation during the driest month shifts to a wetter range (from a range of 4 to 97.5 to a range of 4.5 to 107). In the current potential distribution areas of *T. latifolia*, precipitation seasonality shifts from a range of 8.5 to 97.2 under current climatic conditions to a range of 8.4 to 102.1 under future climatic conditions.

## Discussion

### Why do these Climatic Variables Matter?

Temperature is one of the key factors driving species survival and species distribution. Mean annual temperature has been reported to affect not only plant species assemblage [37], but their distribution as well [38]. However, mean annual temperature by itself is insufficient for species distribution modeling, particularly when considering climate change and the associated variations in temperature rise at various regions. As a result, investigating variations in temperature-related sub-variables as well as the effect of these variations on species distribution can be a means of discovering the consequences of climate change. The effect of mean temperature during the coldest quarter, which is similar to the winter minimum temperature, has been demonstrated to be an important determinant of plant species distribution [39]. The effects of these two variables are clearly demonstrated by our investigation of the impact factor of the potential distribution of *T. latifolia* (Table 1). Generally, increasing these two variables can be unfavorable for the geographic distribution of plant species [40], as confirmed in the present study.

Aside from temperature-related variables, variations in precipitation-related variables and the impact of these variations on species distribution cannot be neglected [41]. It was reported that decreased summer precipitation results in expansion in land areas suitable for invasive plant species (and conversely, increased precipitation leads to habitat reduction) [12]. By contrast, our results demonstrate a different response by invasive species to variations in precipitation. As shown in Table 1, although precipitation of the coldest quarter, mean annual precipitation, and precipitation during the driest month increased under future climatic conditions compared with those under current conditions, this increase does not seem to benefit the geographic distribution of invasive plant species. Contributions of variation in precipitation seasonality (from a narrower range to a broader one) may account for this unfavorable prediction–despite an increase in precipitation, widening of seasonal distribution of precipitation may prove unfavorable for plant species invasion.

### Source of Uncertainties

The future is, by definition, uncertain [42]. As a consequence, our predictions comprise inherited uncertainties from climate scenarios, data, and the manner in which species cope with climate change.

Emission scenario, global climate model (GCM), and initialization of GCM are clearly main sources of uncertainty in global climate projections [43]. First, different emission scenarios reflect different assumptions about development, which are translated into different greenhouse gas emission levels. Second, different GCMs describe climate processes and corresponding feedbacks in various ways. Third, different initialization states make various climate projections to be more or less in phase with actual low-frequency climate oscillations, thus reflecting natural climate variability. Any combination of these sources will generate a future climate that is more or less different from other combinations [44]. A possible way to reduce the uncertainty of climate scenarios might be to adopt the average prediction of future climatic conditions when modeling potential distribution of species.

Uncertainty of data originates from the knowledge that any modeling practice is sensitive to the quality and quantity of employed data, thus species distribution modeling is no exception [42]. Uncertainty of data is related to spatial and temporal resolutions of weather records, reliability and selection of species presence and absence observations, and selection of climatic variables. On one hand, spatial and temporal resolutions of weather records might have an influence on downscaling predictions of GCMs [31]. On the other hand, the finer the spatial resolution of climatic variables is, then the more the micro-climatic features of the climatic conditions could be defined; the coarser the spatial resolution of the climatic variables is, then the more the macro-climatic conditions could be depicted. Second, the reliability of species presence and absence may have an effect on the quantified relationship between species and climatic variables [45]. The selection of presence samples represents the sampling bias of the modeling, and as a result, may influence the prediction of the potential distribution range of subject species. As shown in Figures 3 and 4, the presence records we selected for the modeling procedure are relatively concentrated in Europe and North America. Although such a selection is reasonable for guaranteeing “intact and unbiased sampling”, it may underestimate potential distribution of invasive species at successfully invaded areas because samples in invaded regions are not efficiently used for constructing the relationship between species and environmental variables [46]. Finally, different variables depict environmental conditions in different ways. Climate (such as mean mean annual precipitation, mean annual surface temperature), topography (such as altitude and slope), water availability (such as mean relative air humidity and topographic wetness index), productivity (such as mean annual actual evapotranspiration), human activities (such as distance to the nearest town; [47]), surface condition (such as land use and land cover; [48]), and soil feature (such as fertility, texture and pH; [49]) all have effects on the distribution of invasive species. When selecting variables, the auto-correlation among them needs to be considered. Aside from these abiotic factors, biotic variables also need to be considered [50].

The manner in which a species copes with climate change may be synthesized as evolutionary adaption, dispersal, and extinction [51]. Studies that focused on the response of species to projected climate change assume that climate change outpaces micro-evolutionary processes, and therefore, species have no time to adapt [52]. As a result, the only way for these species to avoid extinction is to move to suitable areas. This finding means that geographic dispersal can be an alternative strategy with which species can maintain reproduction of their populations. In fact, dispersal has been identified as an important response of species to climate change, usually via range shifting to suitable geographic regions. Unfortunately, in the present study, we found that no projected areas are suitable for our target species (*Rumex crispus* and *Typha latifolia*) to disperse to. As such, if these species could not adapt to future climatic conditions, their only destiny would be extinction. By contrast, recent studies have pointed out that evolutionary changes can rapidly take place in several species, especially for species that have invaded new areas [53], [54]. This result indicates that evolutionary adaptation can be an important technique for natural populations to counter rapid climate changes [55].

### Conclusions

In this study, we predicted the potential distribution of two invasive species (*Rumex crispus* and *Typha latifolia*) under current and future climatic conditions by comparing predicted potential distributions. We found that compared with the relatively suitable current climatic conditions, these two species will undergo harsh climatic challenges in the future. In the worst-case scenario, these species will not only lose areas to invade, but will also face possible extinction. We then analyzed possible reasons for such results by extracting important climatic variables and found that warmer and wetter conditions during the coldest season (or month) mainly determine harsh conditions for these invasive plant species. We finally discussed existing uncertainties during our modeling approach. We suggest that climate scenarios, data (climatic variables and species distribution records), and the manner in which species cope with climate change may influence results related to invasion of species, and need to be further investigated in order to reduce the alien species invasion risk under climate change.
